# Pre-and postoperative cerebral blood flow changes in patients with idiopathic normal pressure hydrocephalus measured by computed tomography (CT)-perfusion

**DOI:** 10.1186/2045-8118-12-S1-P59

**Published:** 2015-09-18

**Authors:** Doerthe Ziegelitz, Jonathan Arvidsson, Per Hellström, Mats Tullberg, Carsten Wikkelsö, Göran Starck

**Affiliations:** 1 Neuroradiology, Clinical Sciences, Sahlgrenska Academy at University of Gothenburg, Sweden; 2 Radiation Physics, Clinical Sciences, Sahlgrenska Academy at University of Gothenburg, Sweden; 3 Clinical Neuroscience and Rehabilitation, Neuroscience and Physiology, Sahlgrenska Academy at University of Gothenburg, Sweden

## Introduction

Computed tomography perfusion (CTP) is an established technique, but has not yet been applied to idiopathic normal pressure hydrocephalus (iNPH), in which cerebral blood flow (CBF) is of pathophysiological, diagnostic and prognostic interest. The aim of this work was to determine the pre-and postoperative regional and global CBF in iNPH by CTP, expecting the results to confirm the findings of a perfusion evaluation on the same group of patients and controls, previously performed with magnetic resonance perfusion (MRP).

## Methods

CTP was performed in 18 iNPH patients pre- and 3 months postoperatively. One postoperative CTP was omitted from the analysis because of a confounding subdural hematoma. 6 healthy, age-matched individuals (HI) served as controls at baseline. The CTP covered 4 adjacent 5 mm sections immediately above the posterior commissure. CBF was calculated in 12 cortical and subcortical regions of interest. Besides group comparison of the CBF estimates and examination of individual, postoperative CBF changes, also the correlation of CBF and the severity of symptoms was analyzed. Probable iNPH was diagnosed based on the iNPH Guidelines and clinical performance was assessed according to a newly developed iNPH scale.

## Results

The preoperative CBF in iNPH patients was significantly reduced in the normal appearing and periventricular white matter (PVWM), the lentiform nucleus and the global parenchyma. No CBF differences were found between responders and non-responders. After shunt diversion CBF increased in responders in all anatomical regions by 2.5-32 % to the perfusion level of HI, but remained significantly reduced in the PVWM of non-responders. The pre-and postoperative CBF of cortical and subcortical regions correlated with the intensity of symptoms and the total iNPH scale score.

## Conclusions

In spite of limited spatial coverage, CTP can measure CBF changes in iNPH. CTP confirmed largely previous MRP-based results, indicating the reliability of both perfusion methods.
